# Influence of diabetes mellitus on patients with lumbar spinal stenosis: A nationwide population-based study

**DOI:** 10.1371/journal.pone.0213858

**Published:** 2019-03-15

**Authors:** Chang Kyu Lee, Sun Kyu Choi, Dong Ah Shin, Seong Yi, Yoon Ha, Keung Nyun Kim, Insoo Kim

**Affiliations:** 1 Department of Neurosurgery, Keimyung University Dongsan Medical Center, Daegu, Korea; 2 Department of Neurosurgery, Spine and Spinal Cord Institute, Yonsei University College of Medicine, Seoul, Korea; 3 Department of Medical Research Collaborating Center, Seoul National University Bundang Hospital, Seongnam, Korea; University of Utah Hospital, UNITED STATES

## Abstract

**Purpose:**

To evaluate the relationship between comorbidities, medical cost, and surgical outcome in patients with lumbar spinal stenosis (LSS) and diabetes mellitus (DM).

**Methods:**

Data on patients with LSS (n = 14,298) were collected from the Korean National Health Insurance Service database from 2005 to 2007. After 8 years of follow-up, a “DM group” (n = 3,478) and a “non-DM group” (n = 10,820) were compared according to outcome measures. Cox proportional hazard regressions were performed to examine the relationship between DM, hypertension (HTN), cardiovascular disease (CVD), chronic kidney disease (CKD), cerebrovascular disease (CbVD), and surgery for LSS. The admission rate and medical cost as well asthe overall survival rate for those who underwent lumbar surgery were also assessed among patients with DM and LSS.

**Results:**

Mortality was about 1.35 times higher in the DM group than in the non-DM group. Patients with DM and comorbidities including HTN (hazard ratio [HR], 1.40; 95% confidence interval [CI], 1.25–1.56; p<0.001), CVD (HR, 1.53; 95% CI, 1.36–1.73; p<0.001), CKD (HR, 3.18; 95% CI, 2.7–3.76; p<0.001), and CbVD (HR, 1.69; 95% CI, 1.49–1.91; p<0.001) showed an increased risk of mortality. The mean hospitalization time and average medical cost of patients with DM who underwent lumbar surgery were 60.8 days, and 7,127 USD, respectively. This was 31.3 days longer, and 6,207 USD higher, respectively, than those of patients with DM who underwent conservative treatment for LSS. Within the DM group, the survival rate of surgical management of LSS had a significant tendency for positive prognosis compared with those administered conservative treatment (p = 0.046).

**Conclusions:**

In patients with LSS, DM was associated both with poor prognosis (most significantly in those with CKD), and increased medical cost in those who underwent surgery. Nevertheless, surgical treatment for LSS in patients with DM was related to favorable prognosis compared with conservative treatment.

## Introduction

Diabetes mellitus (DM) has become a major public health problem due to the increasing prevalence of associated morbidity and mortality [1]. The mortality rate of patients with DM is reported to be 2–3 times higher than that of the general population [2,3]. The growth of the DM population is particularly concerning to surgeons, as DM is linked to comorbidities and surgical complications. DM is associated with various chronic diseases, such as hypertension (HTN), cardiovascular disease (CVD), chronic kidney disease (CKD), and cerebrovascular disease (CbVD). DM also increases the risk of heart failure and coronary heart disease [4], and the prevalence and progression rates of CKD are higher in patients with DM [5]. Conversely, in one study, a decrease in cardiovascular mortality was associated with a decrease in ischemic stroke mortality [6]. Moreover, an increased life expectancy and a higher DM incidence lead to an increase in the number of chronic comorbid diseases [5].

Lumbar spinal stenosis (LSS) is also a serious health problem associated with aging. LSS can cause moderate to severe pain, affecting the patient's quality of life, and increasing healthcare costs. Several studies have reported that DM is a risk factor of LSS [7,8], and there is a high prevalence of LSS among patients with DM [9,10]. In studies about the relationship between LSS and DM, one reported that surgical management of LSS in patients with DM was associated with decreased postoperative hemoglobin A1c level, resulting in increased physical activity [11], and another reported that patients with DM had a poorer outcome after spinal surgery than controls, resulting in an increased rate of reoperation and hospitalization [12].

This study aimed to evaluate the relationship between prevalence, mortality, risk of comorbidity, medical cost, and surgical outcome in patients with DM who have LSS, using a large national sample.

## Material and methods

### Database

From 2002 to 2013, this study used data from the Korean National Health Insurance Service (KNHIS) database, which contained data on 1 million nationals who were randomly recruited and nationally represented the entire Korean population. In Korea, almost all people are obliged to enroll in the KNHIS. Approximately 97% of the Korean population are covered by the mandatory health insurance system, and the remaining 3% are under Medicaid, a separate program for the poor. Therefore, the Korean health insurance system holds large databases that reflect the medical situation of the general population. These databases include diagnostic codes, procedures, prescription drugs, personal information of the patient, hospital information, and medical costs. The KNHIS uses the Korean Standard Classification of Diseases (KCD), which was modified from the International Classification of Diseases (ICD).

### Patient selection

**Fig 1:**
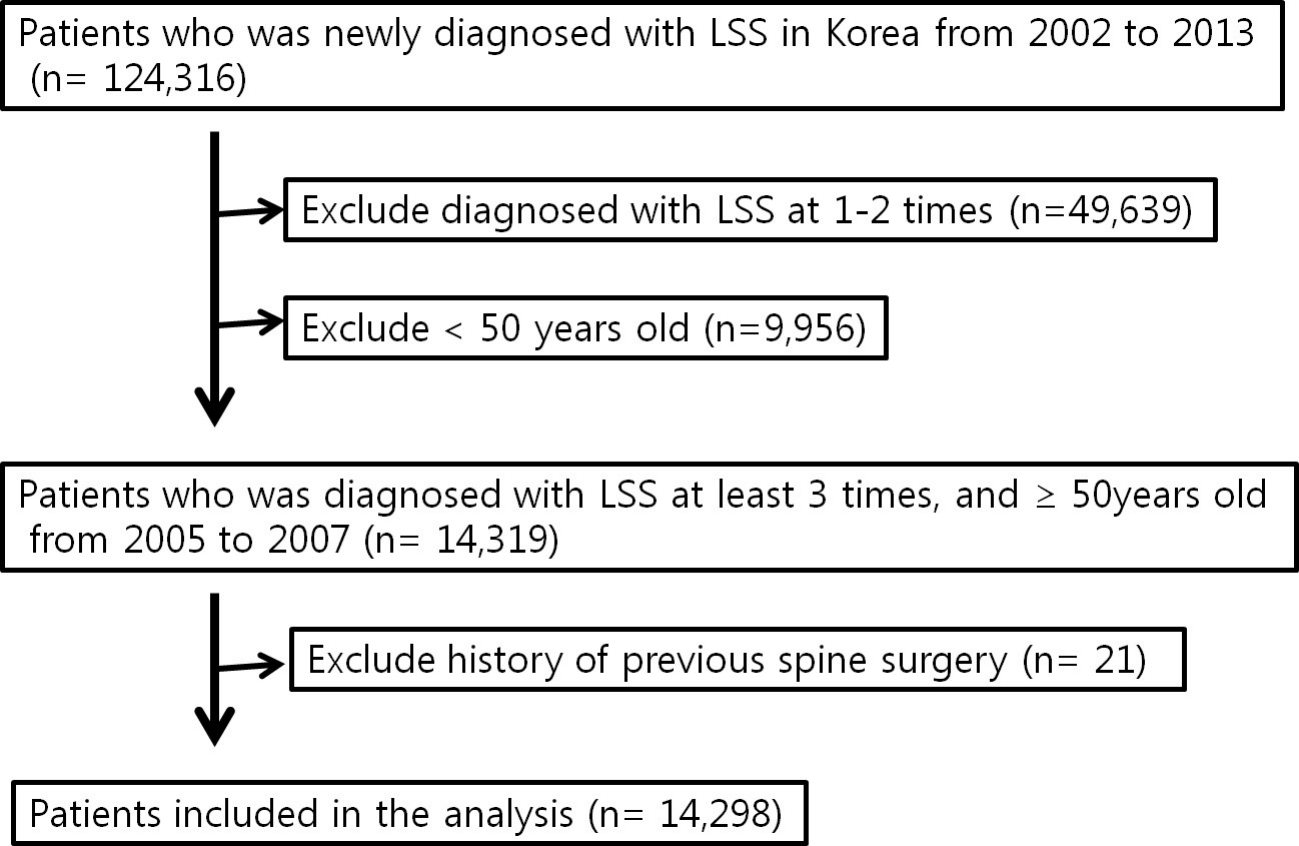
Flowchart of the study population. LSS, lumbar spinal stenosis.

This population-based retrospective cohort study was approved by the institutional review board of Keimyung University Dongsan Medical Center (2018-04-035). The requirement of informed consent was waived by the institutional review board of Keimyung University Dongsan Medical Center. Patients who were diagnosed with LSS in Korea between 2005 and 2007 were selected from the KNHIS database for analysis. The identification of cases is shown in Fig 1. First, we identified patients with a diagnostic code for LSS (M48.0, 48.05, 48.06, 48.07, 48.08, 48.09, 48.8, 48.85, 48.86, 48.87, 48.88, 48.89, 48.9, 48.95, 48.96, 48.97, 48.98, and 48.99). Second, patients who were registered only once or twice for an LSS diagnostic code were eliminated to exclude misdiagnosed patients. Third, we excluded patients who were <50 years old. Fourth, we also excluded patients who underwent a previous lumbar spine surgery. Finally, we selected 14,298 patients (4,778 for 2005; 4,643 for 2006; and 4,877 for 2007) and divided them into two groups (DM and non-DM).

### Variables

Variables included LSS (determined by the KCD codes and diagnosed at least three times), surgical management of LSS (determined by the KCD codes for LSS and operation codes N1499, N2499, N0453, N0466, N0469, N1460, N1466, N1469, N2470, N0444, N0445, N0446, and N0447, which include surgeries such as laminectomy, corpectomy, and lumbar interbody fusion with orwithout screw fixation), and DM (determined by the KCD codes E10-14 and the drug codes for DM). The comorbidity factors were HTN (determined by the KCD codes I10-15 and the drug codes for HTN), CKD (determined by the KCD code N18), CVD (determined by the KCD codes I20-25), and CbVD (determined by the KCD codes I60-69). Other variables included medical cost and hospitalization time for LSS, which reflects economic burden to patients. Medical cost, as total healthcare expenditure, was calculated as the sum of costs to treat LSS, including outpatient clinics, pain clinics, pain medication, hospital admission, etc., for an 8-year follow-up period. Medical cost was calculated in Korean won (KRW) and converted to US dollars (USD) using the average exchange rate for 2015. Hospitalization time was calculated as the sum of hospital admission days for LSS.

### Statistical analysis

Pearson's chi-square tests were performed to examine differences between the DM and non-DM groups. Comorbidities were used to obtain the hazard ratios (HRs) and 95% confidence intervals (CIs) by performing univariate and multivariate Cox proportional hazard regressions. The overall survival rates were calculated using Kaplan-Meier curves for DM, HTN, CKD, CVD, CbVD, and surgical management of LSS subgroups. Surgical outcomes were confirmed using Kaplan-Meier analysis and multivariate Cox regression analysis. All reported P-values were two-sided, and P-values of ≤0.05 were considered significant. Statistical analyses were performed on SAS System for Windows version 9.4 (SAS Inc., Cary, NC, USA).

## Results

The LSS incidence in Korea increased annually, with an overall 1.6-fold increase between 2002 and 2013 (8,693, 8,219, 8,542, 8,932, 8,645, 8,861, 10,011, 10,653, 10,402, 13,614, 13,947, and 13,797 in 2002, 2003, 2004, 2005, 2006, 2007, 2008, 2009, 2010, 2011, 2012, and 2013, respectively). Among the 14,298 patients who met the criteria and were monitored for 8 years, 3,478 patients (DM group) were compared with 10,820 patients (non-DM group). Table 1 shows the characteristics of the DM and non-DM groups with LSS. There was a greater incidence of LSS among women, regardless of DM. The DM group had a significantly higher association with HTN, CVD, CKD, and CbVD than the non-DM group (86.5% vs. 65%, 53.8% vs. 39.8%, 15% vs. 7.6%, and 61.9% vs. 43.8%, respectively). The prevalence ratio of DM in patients with LSS was 24.3%. The overall survival rate of the DM group was 1.35 higher than that of the non-DM group (95% CI, 1.21–1.5; p<0.001).

**Table 1:** Characteristics of the DM group (n = 3,478) and non-DM group (n = 10,820).

Variables	DM	Non-DM	P-value
**Number**	3,478	10,820	
**Mean age (years)**	64.5 ± 8.0	63.5 ± 9.0	<0.0001
**Sex**			0.0084
Male	1,179 (33.9%)	3,420 (31.6%)	
Female	2,299 (66.1%)	7,400 (68.4%)	
**HTN**			<0.0001
No	468 (13.5%)	3,780 (35%)	
Yes	3,007 (86.5%)	7,028 (65%)	
**CVD**			<0.0001
No	1,605 (46.2%)	6,510 (60.2%)	
Yes	1,870 (53.8%)	4,298 (39.8%)	
**CKD**			<0.0001
No	2,953 (85%)	9,982 (92.4%)	
Yes	522 (15%)	826 (7.6%)	
**CbVD**			<0.0001
No	1,324 (38.1%)	6,071 (56.2%)	
Yes	2,151 (61.9%)	4,737 (43.8%)	

DM, diabetes mellitus; HTN, hypertension; CVD, cardiovascular disease; CKD, chronic kidney disease; CbVD, cerebrovascular disease.

**Fig 2:**
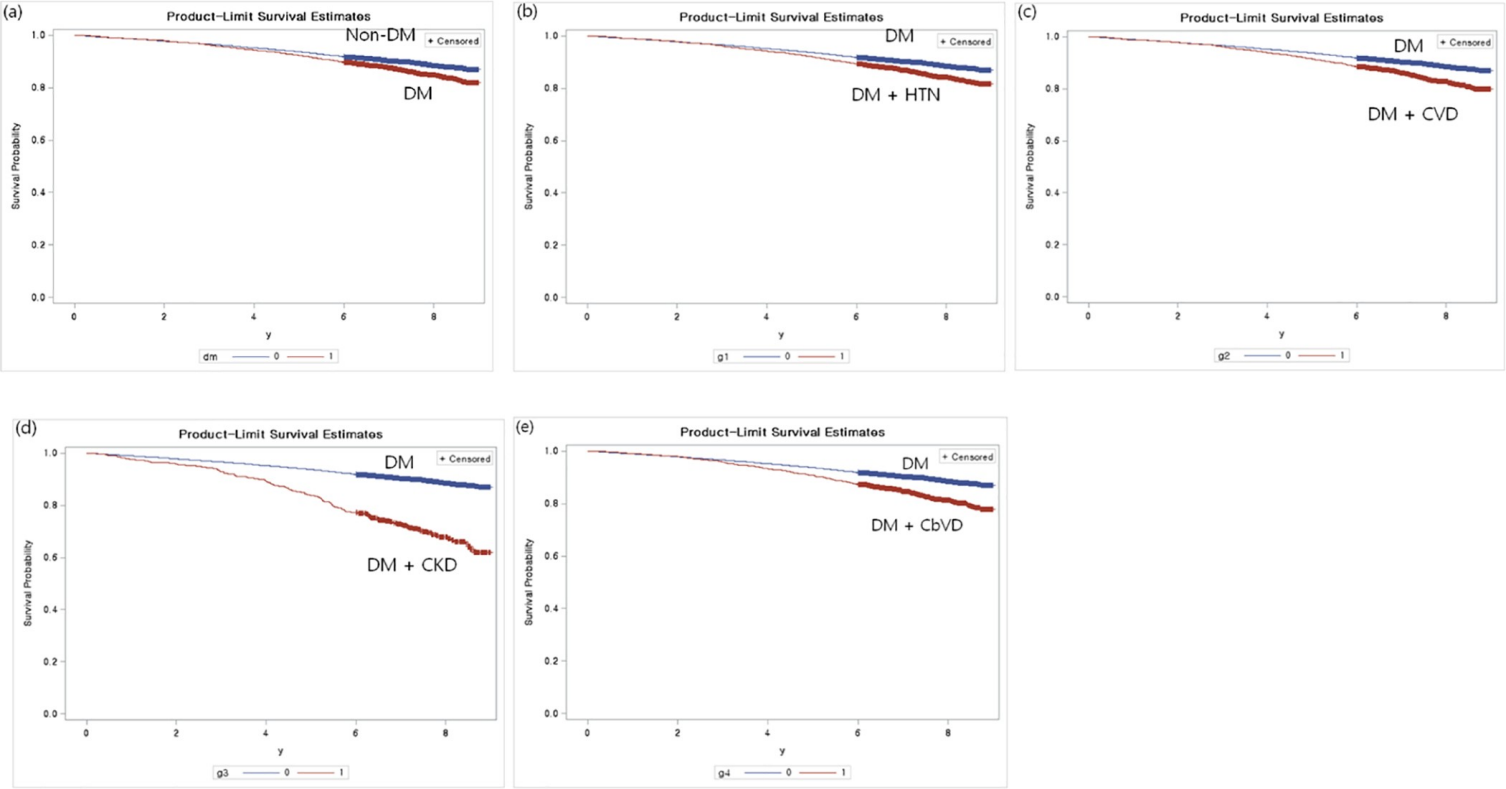
Risk of mortality from DM and comorbidities by Kaplan-Meier analysis. (a) Non-DM vs. DM. (b) DM vs. DM with HTN. (c) DM vs. DM with CVD. (d) DM vs. DM with CKD. (e) DM vs. DM with CbVD. DM, diabetes mellitus; HTN, hypertension; CVD, cardiovascular disease; CKD, chronic kidney disease; CbVD, cerebrovascular disease; vs., versus.

DM with comorbidities significantly increased the risk of mortality compared with DM without comorbidities, based on the results of the Kaplan-Meier survival curves and log-rank tests: HTN (HR, 1.4; 95% CI, 1.25–1.56; p<0.001), CVD (HR, 1.53; 95% CI, 1.36–1.73; p<0.001), CKD (HR, 3.18; 95% CI, 2.7–3.76; p<0.001), and CbVD (HR, 1.69; 95% CI, 1.49–1.91; p<0.001) (Fig 2). Among them, CKD correlated with a higher mortality rate than other comorbidities.

Table 2 shows the comparison of hospitalization time and medical expenses between the DM and non-DM groups. The mean hospitalization time and medical expenses of the DM group were 4.2 days longer (p = 0.0008) and 290 USD higher (p<0.001) than the controls, respectively.

**Table 2:** Comparison of length of hospital stay and medical expenses between the DM group and non-DM group in patients with lumbar spinal stenosis.

	Number	Hospitalization (mean no. of days) (min-max)	P-value	Medical expenses (average in USD) (min-max)	P-value
**DM**	3,386	34.9 (1–1,318)	0.0008	1,590 (4–49,021)	<0.001
**Non-DM**	10,574	30.7 (0–1,336)		1,300 (6–108,060)	

DM, diabetes mellitus; USD, United States dollars.

**Fig 3:**
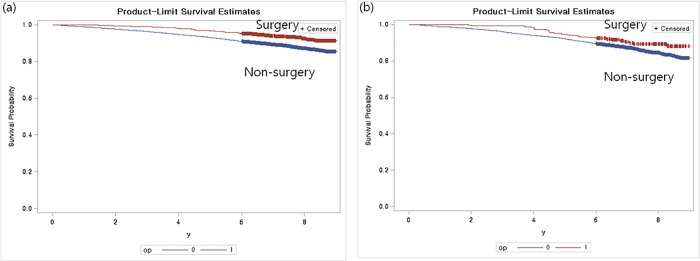
Surgical outcomes in (a) LSS and (b) LSS with DM by Kaplan-Meier analysis. DM, diabetes mellitus; LSS, lumbar spinal stenosis.

In the DM group, surgery ("surgery group") and no surgery ("non-surgery group") for LSS were compared in terms of survival rate for prognosis, hospitalization time, and medical expenses. Patients with LSS were significantly associated with favorable surgical outcomes (p<0.0001). Additionally, patients with DM who underwent surgery for LSS had a significantly better prognosis than those with DM who underwent conservative treatment (p = 0.046) (Fig 2). There was a 31.3 day difference in hospitalization time between the surgery and non-surgery groups, and medical expenses showed a difference of 6,207 USD between the groups (7,127 USD and 920 USD, respectively; p<0.0001; Table 3). We compared the surgical outcomes in LSS and LSS with DM using Kaplan-Meier analysis and multivariate cox regression analysis. Fig 3 reveals that the survival rates for LSS and LSS with DM in the surgery group were improved compared with the non-surgery group (HR, 0.55; 95% CI, 0.43–0.7; p<0.001, vs. HR, 0.68; 95% CI, 0.46–1; p = 0.046). In patients with LSS, conservative treatment showed a 1.91-fold higher mortality rate than surgical treatment, and a 1.61-fold higher mortality rate in the DM group even after adjusting for all comorbidity factors (95% CI, 1.5–2.44, p<0.001, and 95% CI, 1.09–2.37, p = 0.017, respectively; Table 4).

**Table 3:** Comparison of length of hospital stay and medical expenses between the surgery and non-surgery subgroups of the diabetes mellitus group.

	Number	Hospitalization (mean no. of days) (min-max)	P-value	Medical expenses (average in USD) (min-max)	P-value
**With surgery**	268	70.5 (1–956)	<0.0001	8,017 (8–49,305)	<0.0001
**No surgery**	3,118	31.8 (1–1,318)		1,048 (4–38,712)	

USD, United States dollars.

**Table 4:** Multivariate Cox regression analysis of variables according to risk of mortality in LSS and LSS with DM.

Variables	Multivariate Cox (LSS)			Multivariate Cox (LSS with DM)		
	HR	95% CI	P-value	HR	95% CI	P-value
**Group**						
Non-DM	1 (reference)					
DM	1.12	1.0–1.25	0.049			
**Treatment**						
Surgery	1 (reference)			1 (reference)		
Non-surgery	1.91	1.5–2.44	<0.001	1.61	1.09–2.37	0.017
**HTN**						
No	1 (reference)			1 (reference)		
Yes	0.9	0.8–1.02	0.09	1.05	0.78–1.42	0.73
**CVD**						
No	1 (reference)			1 (reference)		
Yes	1.64	1.48–1.82	<0.001	1.52	1.25–1.85	<0.001
**CKD**						
No	1 (reference)			1 (reference)		
Yes	2.83	2.51–3.19	<0.001	2.81	2.31–3.41	<0.001
**CbVD**						
No	1 (reference)			1 (reference)		
Yes	1.23	1.11–1.37	<0.001	1.16	0.95–1.42	0.14

DM, diabetes mellitus; LSS, lumbar spinal stenosis; HR, hazard ratio; CI, confidence interval; HTN, hypertension; CVD, cardiovascular disease; CKD, chronic kidney disease; CbVD, cerebrovascular disease.

## Discussion

This retrospective cohort study investigated the relationship between DM and LSS using a national database. We confirmed that among patients with LSS, the DM group had a 1.35-fold higher mortality rate than the non-DM group. There was a higher risk of mortality when DM occurred with comorbidities such as HTN, impaired renal function, and vascular diseases. Furthermore, we found that patients with DM with surgical management of LSS had a better prognosis than those with DM who underwent conservative treatment.

### LSS with DM vs. without DM

The prevalence of DM has been continuously increasing over time. Abraham et al. reported that the annual rates of DM per 1,000 individuals were 2.6, 3.8, 4.7, and 3.0 for women and 3.4, 4.5, 7.4, and 7.3 for men in the 1970s, 1980s, 1990s, and 2000s, respectively [13]. Patients with DM were estimated to account for 8.8% of the world's population, and the International Diabetes Federation predicts that the DM population will rise to 642 million by 2040 [14]. In Europe, morbidity from DM among the adult population is reported to be 8–10% [15]. The World Health Organization estimated that 1.5 million deaths were directly caused by DM in 2012 and that 5 million deaths were attributed to DM in 2015 [14]. DM is a chronic disease affecting the connective tissues, including the bone and cartilage [16,17], peripheral nervous system, blood vessels, and intervertebral discs, causing early degeneration [18,19]. Therefore, DM may be related to the pathogenesis of spinal stenosis [20]. In this study, 24.3% of patients with LSS had DM, which is about 2.8-fold higher than the average DM population. Moreover, among patients with LSS, the DM group showed a significantly higher overall survival rate than that of the non-DM group.

### DM and comorbidity

The mortality rate of patients with DM is reported to be about twofold higher than that of the general population [2,3]. Additionally, some studies reported a tendency of risk of disease progression when DM occurs with CVD (including stroke, myocardial infarction, angina pectoris, heart failure, ischemic heart disease, coronary heart disease, atherosclerosis, and cardiovascular death) and CKD [5,21]. In our study, DM with HTN, CVD, CKD, or CbVD showed a significantly increased mortality rate, especially with CKD. Moreover, as we selected patients with DM who had LSS and were aged ≥50 years, we can assume that poor glucose control and low physical activity, due to neurogenic intermittent claudication and radiating leg pain, both lead to risk of progression of comorbidities with DM. Einarson et al. reported that CVD affected about 32% of the DM population, with coronary artery disease and stroke as the major causes of mortality [21]. There has been also an increasing population of older DM patients with CKD due to long life expectancy and high DM incidence [22]. According to the National Health and Nutrition Examination Survey (NHANES), the prevalence of CKD in older patients with DM increased from 27.3% during 1988–1994 to 40.6% during 2009–2014 [23]. While the progressive decline in glomerular filtration rate (GFR) normally accompanies aging, DM accelerates the GFR decline, leading to CKD development [24]. Furthermore, in patients with DM, CKD increases cardiovascular morbidity and mortality and is associated with renal replacement therapy [25]. Thus, DM is related to various comorbidities, affects their progression, and increases mortality.

### Hospitalization time and medical cost

As DM prevalence is increasing, medical expenses for DM also increase. Jacobs et al. reported that people with DM had 1.7-fold higher health expenses than people without DM [26]. Meanwhile, LSS occurs with aging, and its prevalence is increasing with older age. Lee et al. [27] reported an increased incidence of spinal diseases with an increase in the aging population, with a peak incidence rate of 42.6% in the age group 75–79 years. Between 2012 and 2016, the medical costs for spinal stenosis comprised the largest portion of expenses for spinal diseases in patients aged >60 years [27]. In our study, we divided the patients with LSS into the DM and non-DM groups, and compared their total lengths of hospital stay and medical expenses, which were only associated with LSS after 8 years of follow-up. We found that LSS with DM was significantly associated with longer hospitalization and higher medical cost. Furthermore, hospitalization time and medical expenses were greater in the surgery group than in the non-surgery group. Hence, DM and LSS significantly increased the healthcare burden of the patients.

### Surgical outcome of LSS patients with DM

Surgical treatment of LSS has been consistently reported to bring about reliable positive results [28,29]. However, DM is closely associated with postoperative surgical site infection, leading to a worse prognosis. Fei et al. reported that DM is the most important predictor of surgical site infection after lumbar spinal surgery [30]. Moreover, recently, surgical management of LSS demonstrated questionable benefits compared with conservative treatment, with the rate of side effects ranging from 10% to 24% in surgical cases and no side effects for conservative treatment [31]. Here, in the DM group, surgical management of LSS was significantly associated with better prognosis, and we found similar results in the total LSS population after adjusting for comorbidities. In contrast to previous studies, this study produced meaningful results on surgical outcomes, which were possible through using operational definitions to reduce bias, using a large national sample, and performing a long-term observation.

### Limitations

This population-based study has some limitations. First, we used insurance claims data, which lacked information about the patient's laboratory findings and lifestyle factors related to DM, and nonreimbursable items, which included nonsurgical treatments, procedures, and medicines not covered by the Korean health insurance system. Second, the medical expenses could be inaccurate due to the type of populations and diagnostic codes included by the insurance claims. Only medical expenditures by beneficiaries of KNHIS were covered, whereas those with Medicaid were not. Moreover, the medical costs were calculated from insurance claims stating LSS as the “main diagnosis” and “second diagnosis”. For these reasons, there was possibility of underestimating medical expenses. Nevertheless, we used operational definitions for LSS, DM, and comorbidities to improve diagnostic reliability.

## Conclusions

Patients with DM who had LSS showed a poor prognosis when DM occurred with HTN, CVD, and CbVD, and most significantly with CKD. Although LSS with DM was negatively associated with hospitalization time, medical cost, and mortality, surgical treatment for LSS in patients with DM was associated with a favorable prognosis compared with conservative treatment.
